# Impact of Clinical Video Scenarios Used for a Summative Exam to Facilitate Learning

**DOI:** 10.1111/eje.13050

**Published:** 2024-12-02

**Authors:** Michael George Botelho, Bochra Boubaker

**Affiliations:** ^1^ Faculty of Dentistry The University of Hong Kong Hong Kong China

**Keywords:** clinical skills, dental studies, learning dialogue, skills, summative assessment, undergraduate curriculum, vicarious learning, video

## Abstract

**Introduction:**

This article explores the use of clinical vicarious learning dialogue videos as a learning resource for a written summative assessment.

**Method:**

A prescribed list of 42 clinical vicarious learning dialogue videos was disseminated to students, and they were informed that these would form the scope of a prosthodontics question in their final year summative exam. The videos captured the learning dialogue between a teacher and student during diagnosis, problem‐solving or clinical decision‐making in relation to prosthodontic patient interactions. Exam questions were created from screen capture images from the videos based on and around the video content. After the exam, video analytics was captured, and students were invited for an interview using a question guide which was recorded and transcribed and a thematic analysis was performed using a deductive inductive approach.

**Results:**

Fourteen students were interviewed, and from these three domains and 10 key themes were identified: **learning**: learning strategy, learning new skills and knowledge, learning clinical skills, application of learnt skills and engagement; **assessment**: exam scope and stress, clinical relevance, motivation, generalisable; and **video as a learning medium**: enjoyable, affordance. Students reported that using these videos facilitated and supported their exam preparations, stimulated learning new content as well as higher‐order thinking skills. Students reported they had applied skills learnt from the videos and broadened their cognitive skills and practical experience. The format of the assessment was described as enjoyable and reduced stress. All students reported they watched ‘all’ the videos which appeared to be supported by the analytics.

**Conclusion:**

Clinical vicarious learning dialogue videos were found to help learning, assessment literacy, clinical cognitive skills, stress and motivation for learning.

## 1 Introduction

Assessments are an important part of determining progress and fitness to progress in clinical training. However, written exams are often used in this process, and they do not easily correspond to the ‘clinical skills’ students need to support clinical competence. While OSCEs have been used for many years in healthcare to assess students' clinical skills with a psychomotor focus, these are time and resource intensive and alternative assessment should be explored, especially those examining cognitive clinical skills.

A delineation of what clinical skills comprise for assessments is important to determine how best to assess them. In essence, clinical skills comprise cognitive, psychomotor and combinations of these attributes. While OSCEs or simulation laboratory tests in dentistry have traditionally focused on psychomotor skills performances, there is also a need to examine clinical cognitive skills with regard to higher‐order thinking skills such as diagnosis, problem‐solving and clinical decision‐making. There is a need to support students' clinical transition as they move from simulation laboratory to patients or learning new skills. Students have reported significant stresses during this clinical transition stage which has been attributed to different domains such as a lack of knowledge and skills including ‘soft skills experience’ in connection with diagnosis, treatment planning, problem‐solving and patient communication [1, 2].

Traditionally diagnosis, problem‐solving and clinical decision‐making have been learnt during clinical practice and such clinical exposure, however, an issue with this is learning is not uniform across students during their clinical training. This relates to the nature of the clinical learning environment and patient needs which means it is not possible to present content in a learner‐centric manner and this is a limitation of authentic real‐world learning. However, aspects of this could be addressed and supported by authentic learning experiences and assessments to help with these cognitive skills. Therefore, the assessment of these higher‐order thinking skills needs to have appropriate assessment methods, not only that but these should be aligned to appropriate learning content for these assessments.

Video has become an increasingly important tool for learning across a range of disciplines and in particular in healthcare [3]. This has been used for facilitating learning basic science such as anatomy [4] and clinical skills like clinical examination [5], endocrinology [6] or anaesthesia in dentistry [7]. While video content has traditionally been used as an instructional tool for knowledge dissemination or skills performance focusing on the psychomotor, a new modality has explored chairside clinical dialogue based on diagnosis, problems or clinical decision‐making. This new type of video genre has been described as vicarious learning dialogue video [8].

Learning dialogue is considered the exchange between teacher and students as a teacher explores a student's understanding of a clinical issue or problem through iterative questioning to resolve the issue [9]. These clinical learning dialogue interactions were video recorded to capture clinical decision‐making or problem‐solving which have been archived for peers to learn from on a learning management system and therefore comprise a vicarious learning experience that can be curated and watched on demand. This library of clinical videos is considered as ‘synthetic clinical experience’ in that students can learn clinical cognitive skills before they go to clinics. These videos have been reported to be helpful for remembering knowledge and stimulating students to think as well as reducing stress, improving confidence and better preparedness for clinical assessments [10]. However, while these vicarious learning dialogue videos were reported to be well regarded by students, it was apparent that not all students accessed the video learning materials prior to clinics.

Therefore, a strategy was designed to both engage students with the video library content and create a new type of assessment that evaluates students' higher‐order thinking skills. Screen capture images from these videos were used to create questions in a summative written assessment. The intent was to drive students to consume the content, learn new knowledge and skills from the videos that support: diagnosis, problem‐solving and clinical decision‐making.

The aim of this study was to perform a qualitative analysis of students' experience with this novel assessment approach based on defined clinical video content and how they responded to this and examine the analytics of their consumption.

## 2 Methods

### 2.1 The Assessment

The question for the assessment was designed by the lead author based on a list of 42 videos in four different categories. These related to resin‐bond bridges, crowns and fixed partial dentures, aesthetics and tooth wear and were mapped to the course learning outcomes. These videos captured the chairside vicarious learning dialogue between teacher and student associated with problem‐solving, diagnosis and clinical decision‐making [11, 12, 13] that were recorded from other students' treatment sessions.

**FIGURE 1:**
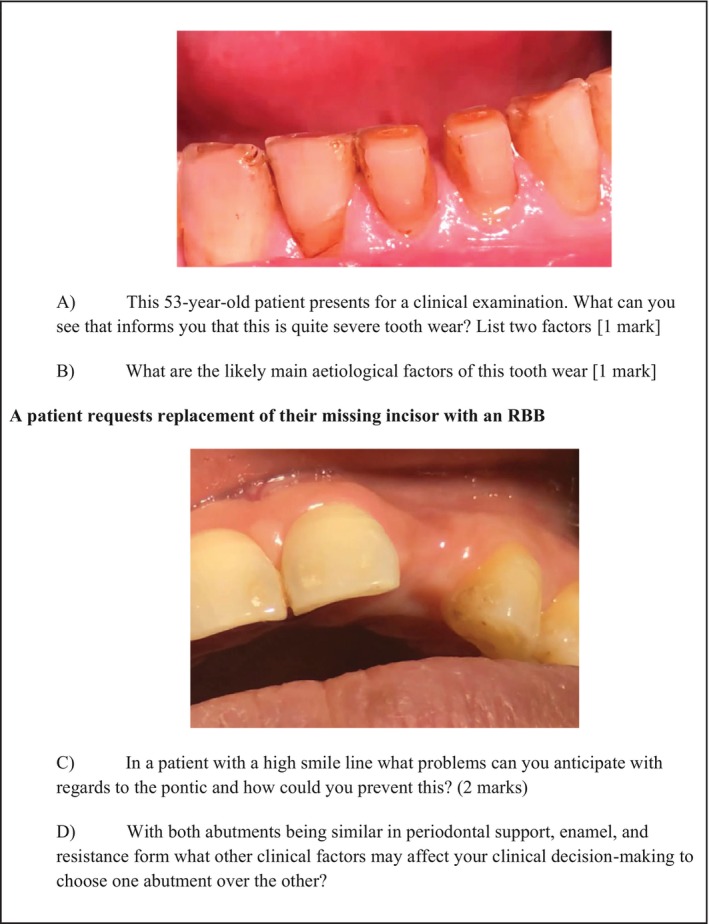
Sample exam questions.

The exam comprised 10 discipline‐based sections, split into two papers, with each paper lasting 3 h. Each discipline‐based section was weighed 20 marks each. For the prosthodontics section, 10 questions were devised based on seven photographs from the vicarious learning dialogue videos. The authors generated these questions by using screen captures from the videos, addressing topics covered in the videos as well as related problem‐solving, decision‐making and procedural knowledge associated with the video content (Figure 1).

Students were sent an email, 6 weeks before the exam, notifying them of the scope of the exam with the list of the videos and URL links they should watch and that the exam would be based on content from these videos (Appendix S1).

### 2.2 Participants Recruitment

A message was sent, a few weeks after the exam, individually to all 75 of the BDS six students by e‐mail to participate in an interview. A coffee voucher was offered as an incentive to participate. Initially, 15 responses were obtained, and 14 interviews were scheduled allowing a convenient sampling up to saturation.

### 2.3 Data Collection

A question guide for interviews was prepared and reviewed by the course academic lead and the research interviewer to explore students' experience of the exam notification, self‐study revision, and written examination and their associated perceptions. The questions covered four key areas relating to: video consumption, video's role in the learning process, assessment and application of knowledge (Table 1). A set of 24 guiding questions was developed to outline the structure of the interview, allowing for additional follow‐up questions when necessary. Analytics of videos watched was downloaded in an Excel file, and a frequency distribution was performed based on time and each video. Ethical approval for the study was obtained from the Institutional Review Board of The University of Hong Kong (reference no. UW 19–270). This study follows the COREQ guidelines and is reported in Appendix S2.

**TABLE 1:** Structured interview questions that were used as stimuli for discussion to explore students' perceptions.

Guidance interview questions
Video's consumption
Did you watch the videos before the exam?
How many of them did you watch?
Did you watch some videos more than once?
Did you watch them because you knew the exam would be based on them?
Did you watch alone or with peers?
Did you watch them all in one session or more?
Did you watch at a particular time of day or setting?
Were you aware of these videos before the exam? Have you watched any before?
Was watching the videos enjoyable or challenging?
Video's role in learning process
Were these videos useful? How did these videos help you?
What was your learning strategy/revising when watching the videos? Did you take notes or screen captures?
Did watching these videos help with any new learning? Do you remember what you learned that was new?
Were you confused by any of the content in the videos?
Did the videos stimulate you to search for new content or revise old?
Did these videos help with clinical decision‐making, problem‐solving or clinical skills? To what extent?
Assessment
Do you think this exam is useful to your learning?
Do you think other clinical subject areas could use this approach?
Would you say that this type of exam was difficult or easy for you?
Did you think about what questions you may be asked on the content of the exam?
Were you surprised by any questions in the exam or found them not relevant to the video content?
Do you think this kind of exam facilitates assessment of your problem‐solving ability accurately?
If you had the choice, would you prefer the exam you had, or a more classic exam? Why?
Several weeks after the exam, what remains as knowledge and acquisition from these videos?
Application of knowledge
Have you applied what you learned from these videos in your clinical learning, or do you think it could be useful to you in the near future?

An impartial female researcher interviewed all the students individually who had clinical and higher training (DDS and MSc) and was not involved in the course, assessment or delivery of content and was unfamiliar to the students before the interviews. The interviews were conducted in a quiet office room at the dental faculty. It was conducted face‐to‐face in English without interruption lasting between 13 and 25 min. No one else was present besides the participant and researcher. The nature of the interview was explained to the student and that all information would be anonymous. It was emphasised that there were no right or wrong answers and that they were invited to elaborate on their answer. Informed consent was signed by each participant. No students dropped out after attending the interview.

### 2.4 Data Analysis

The interviews were audio recorded and then transcribed automatically using a readily available and cost‐effective transcription software, Otter.ai (Otter.ai, USA), after which the transcriptions were manually corrected after replaying the recordings. Initially, 12 interviews were transcribed, and upon conducting a content analysis, it was determined that data saturation had been reached. To confirm the saturation, the next two interviews were listened to, and no new data was collected. Once all the data was transcribed, a thematic analysis was carried out as described by Nowell et al. [14] which involved six phases:

1. Familiarisation with the data: involved the interviewer listening to all the audio recordings twice to confirm accuracy which was then followed by active engagement of reading of the data transcripts, with the aim of identifying meanings and patterns within them.
2. Generating initial codes: an Excel spreadsheet was used to log all the transcript data which was analysed line by line with keywords that were allocated to key phrases or words. Initial codes were generated using an inductive approach, which involved identifying codes based on how they emerged from the data.
3. Searching for themes: through the process of coding, substantive codes with similar content were identified and clustered into themes.
4. Reviewing themes: the data were reviewed by the lead author and the themes were clarified and confirmed through further discussion until a consensus on the interpretation was achieved to determine the most appropriate theme.
5. Defining and naming themes: analysis of the themes was performed to group them into higher broader domains based on similarity using an interactive approach to refine themes and sort into domains.
6. Producing the report: the key components of each domain and associated theme were outlined and supported by original quotes taken directly from the transcripts.

An embedded mixed methods approach was used with regard to the video analytics and responses to questions related to students' learning strategies.

## 3 Results

From the thematic analysis of the transcripts list three domains and 10 themes were derived (Table 2).

**TABLE 2:** Main domains and themes identified.

Learning	Learning strategy
	Learning new skills and knowledge
	Learning clinical skills: Broadening experience Learning dialogue and cognitive skills Psychomotor skills
	Application of learnt skills and engagement
Assessment	Exam scope, stress and knowledge level
	Clinical relevance
	Motivation
	Generalisable
Video as a learning medium	Enjoyable mode of learning
	Affordance

### 3.1 Learning

#### 3.1.1 Learning Strategy

**FIGURE 2:**
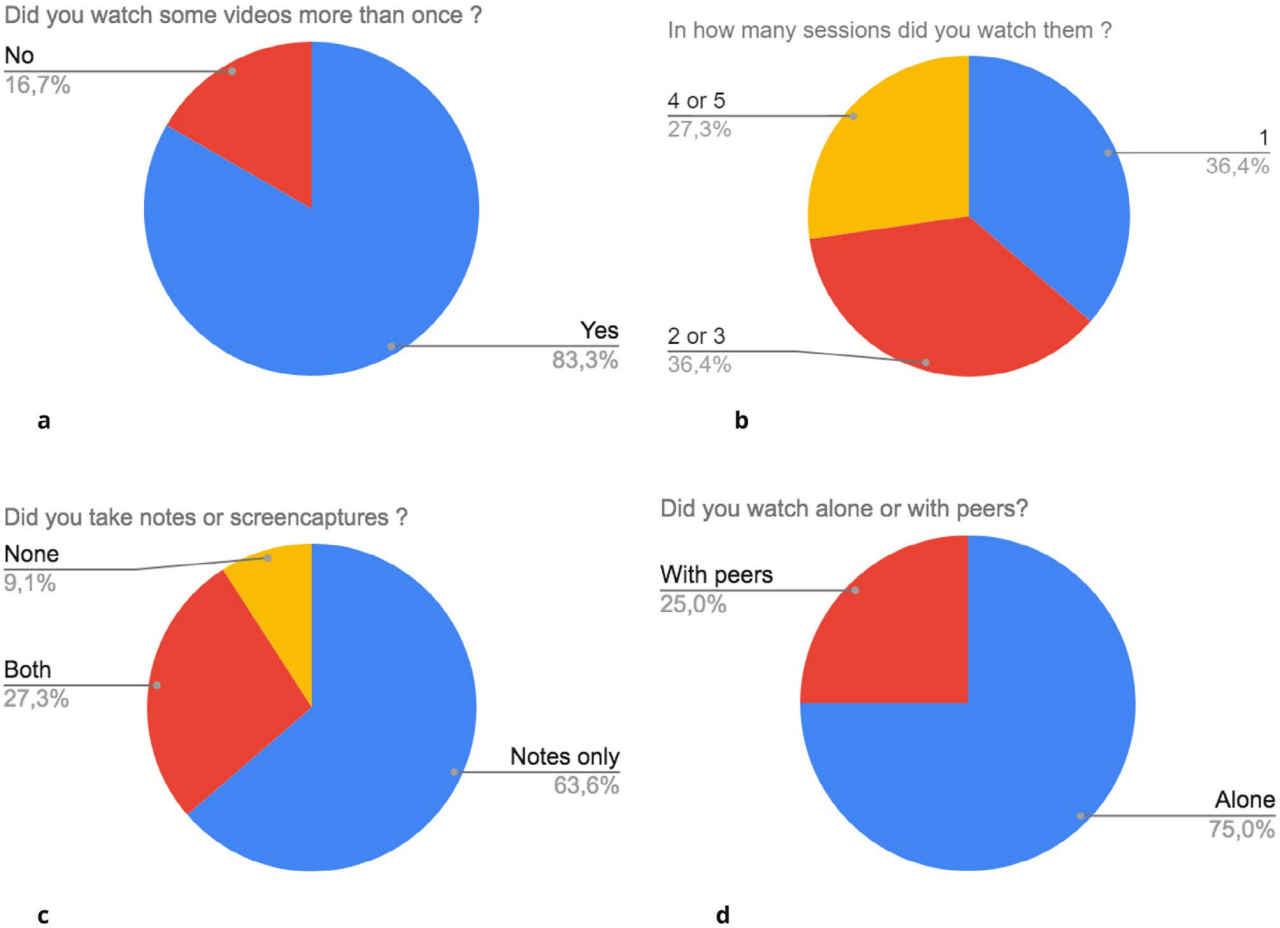
Quantitative data derived from the analysis of qualitative data regarding students' learning strategy.

Here we define the learning strategy as the approach and techniques that students used in order to learn from the videos for their assessment. After receiving an email indicating the exam scope and list of video URLs, the vast majority of the students reported watching all the videos more than once over two or three sessions; this was confirmed by the analytics (Figure 2a,b).

Ninety per cent of students reported taking notes and a few stated they performed screen captures from the video to annotate their notes (Figure 2c). Students reported different approaches to learning from the videos with regard to note‐taking and how they watched the video.

I watched each one and (…) I would finish watching the whole video without writing anything down. Because I think writing doesn't help with understanding. I tend to watch everything first. And then based on my memory immediately after watching the video I write down … only write one line in my notebook for each video because it's very concise (Student 4).

Students also reported to

think about what kind of questions might pop up in the exam. And I write them down and right before the exam, I watched the videos for the second time just to refresh my memories (Student 2).

Twenty‐five per cent of students reported watching the videos with peers as it helped learning, was enjoyable and stimulated discussion with peers (Figure 2d). Watching with peers helped in preparation for the exam so they ‘can share our knowledge when we watch together and … it's less boring’ (Student 12). Students reported making notes with peers and interacting;

we read them back again and see if they have any doubts and just discuss with my friends (Student 1).

Because it's always good to ask each other questions and … we have peers to study together, I think we have better memory. We talk about the materials, talk about the content, and then when we have something not sure then we can ask for others assurance or clarification (Student 5).

#### 3.1.2 Learning New Skills and Knowledge

Students reported learning diagnosis, new skills and content knowledge from watching the videos in particular:

… it helps … how to distinguish what kind of tooth wear needs to be restored or how to manage them … [in particular videos on] microabrasion … because I haven't done that in my clinical sessions. So that helped (Student 2).

Also, ‘the putty and wash tricks are also new for me because I don't really use putty and wash [impression]’ (Student 1) and ‘the marginal ridge and the plunging effect of the cusps that was new to me’ (Student 2).

However sometimes the content was perceived to be confusing as in two videos, different procedures were recommended or there were issues with the sound quality. In particular, different cementation approaches to bridges were mentioned ‘So it's a bit confusing …but … I ended up having to make my own judgment on what to do when I had to do this clinically’ (Student 4).

Students reported an advantage of the clinically situated video content compared to simulation laboratory learning being practical and with authentic clinical contexts, also some of the video content was new to them and helped fill in gaps in their clinical experience. ‘I think it adds more knowledge to the basic knowledge’. They highlighted that ‘there's a limit to the sim lab course … it cannot teach you everything …’ ‘The videos are recorded in the clinic, so they are more practical and more like there are real cases that we can study on. So some of those situations were not explained in a sim lab and videos will add the … missing parts we need for actual practice’ (Student 12).

Students also indicated that the vicarious learning dialogue videos encouraged them to search for new content and discuss with peers.

When I watched the videos, I tried to think outside the box. What else I can learn … Like for example, … he was talking about electrosurgery … it's something that I've never really done in class before. So … I was able to look more into it in depth (Student 9).

The clinical videos also stimulated students to ‘discuss with my classmate after watching some [RBB] designs … and then list the reasons why the pros and cons of each design so and also search for some material’ (Student 11). However, some students felt that the content already provided quite detailed information, so they did not feel the need to search for additional information or new content.

#### 3.1.3 Learning Clinical Skills

Here we consider clinical skills to include both psychomotor skills and cognitive skills. These cognitive skills include but are not limited to diagnosis, critical thinking, problem‐solving, clinical decision‐making and treatment planning and may be considered as higher‐order thinking skills.

##### 3.1.3.1 Broadening Clinical Experience

The clinical videos facilitated students to be exposed to a range of different clinical cases and complexity of their peers and the associated learning dialogue. This not only afforded a broader and deeper learning experience through observing peers' cases but also observing peers in their learning experience.

As a dental student, we don't have exposure to a wide variety of cases … but through the videos you can learn from your classmates' cases (Student 4).

The [course] manual cannot include all the cases and all the scenarios … but in the video there are different cases, so we have to learn how to plan the treatment for different cases (Student 6).

Clinically, it includes many cases that I haven't met before and some of them are more complicated than my own patients. So I may have a more deeper understanding or, what if, I meet the similar cases in the future (Student 11).

They found it ‘helpful clinically, like you learn skills from other people's cases. It's like you have another patient to learn from’ (Student 2).

##### 3.1.3.2 Learning Dialogue and Clinical Cognitive Skills

The learning dialogue was a key component of the clinical videos. Learning dialogue has been described as a ‘dialogue that includes a learning episode, discussion of a problem with a tutor or peer student, in which an impasse is resolved, a concept clarified or a solution explained’ [9].

These videos were reported as being novel in supporting clinical cognitive skills ‘I think what's interesting is the discussion between the student and professor XXX. Some of the discussion content we may not be able to get from books, because it's a clinical judgment thing. From my point of view, I think the only source of such information that could help develop my critical thinking is through these videos or in the clinic’ (Student 4). Students also reported the videos' role in developing their treatment planning skills.

The videos focus on the treatment planning not only on the clinical skills, but also the planning part. So I have learned more about … how should we plan for the patient if we encounter different scenarios (Student 6).

#### 3.1.4 Psychomotor Skills

According to the students, certain content was especially beneficial for learning, applying or improving psychomotor skills. ‘Some really practical points in the clinic [videos]. I think they are … quite useful, … like taking an impression or checking an occlusion … so we can just learn from those procedures that are recorded … so I tried to do it again.’ (Student 3).

Students also reported that ‘… some of the techniques were new to us’ (Student 5). ‘For example, how to perform microabrasion and, when to perform that and … understand the extent of the tooth wear and how to manage them’ (Student 2).

#### 3.1.5 Application of Learnt Skills and Engagement

Students reported that the videos for the exam were beneficial for facilitating their skills application or problem‐solving in future situations: The videos are ‘quite practical’ (Student 5) and ‘it gives us a framework of how, what things we can consider and how we can plan a treatment for patients’ (Student 9). Students also believed that the information from the videos would be recalled during clinical encounters, as one student expressed, ‘I will recall … the information in the videos when we see some cases clinically’ (Student 5). Another student reinforced this sentiment, saying: ‘like next time when you see a similar case … I can use more or less similar, like management strategies’ (Student 2).

Students demonstrated active engagement with their learning material, extending beyond the upcoming exam. A student reported ‘even after getting this email (exam scope), I watched a few more that weren't in here because I found the other ones interesting. So I clicked onto them.’ Furthermore, students reported watching similar vicarious learning dialogue videos ‘Before getting this email, … not all but some of them which were clinically relevant to what I was going to do in a particular session,’ reporting ‘it would help me have a better idea of what to do on the clinic’ (Student 4), suggesting that students viewed these videos to deepen their understanding and apply knowledge for clinical practice.

### 3.2 Assessment

#### 3.2.1 Exam Scope and Stress

The exam was generally considered ‘fair … but I wouldn't say it's easy either’ (Student 4). For most of them it was easier compared to other disciplines' questions because students had the videos to watch and also it is related to cases that are relevant and frequently seen in clinics.

Firstly, because Prof XXX gave us these videos to watch. And second, I think like those pictures are usually the problems or the cases that we can see in clinic. So we have more experience or more knowledge about them. But we've been talking about oral surgery OMFS like those questions are quite difficult because we are seldom encountered those cases, like cancer and anything else (Student 7).

Students reported having ‘a lot of stress and pressure studying for final exam. We have to study all the contents from year one to year six’ (Student 5). Knowing the scope of the exam created the possibility of focusing on the elements to be reviewed and being less stressed and more confident:

First of all, I have less stress because I've seen something similar based on the videos … I feel more confident that I will be able to write something down at least (Student 4).

On the other hand, such an approach may limit learning by studying less broadly.

if every subject is like this, then probably I would not study as much … once you've been given this kind of clues of what's going to be in the exam, you sort of just focus that area and sort of neglects the other part (Student 2).

Students also regarded this exam as well‐designed with regard to levels of knowledge assessed.

I think this is very well‐set exam … some of the questions, they are recall, but there are other ones which are not just recall, you have to watch videos you actually have to understand it and then apply, express the content in your words and apply really … So it helped reinforce my understanding based on the videos (Student 4).

#### 3.2.2 Clinical Relevance

Students perceived the assessment based on videos as clinically relevant and interesting:

The exam is more focused on the clinical cases rather than only the knowledge or theory (Student 6).

There is a wide variety of topics tested here and they're all clinically relevant. So this is interesting. I prefer this to something which is more theoretical, boring, but these ones are interesting (Student 4).

#### 3.2.3 Motivation

Even though some students were aware of the existence of these videos before the exam's scope was announced, they did not watch all the clinical videos; however, the assessment incentivised them to engage with the content.

We didn't always check everything … but before the exam we will really check more … we got more motivation when we know that there will be an exam (Student 5).

**FIGURE 3:**
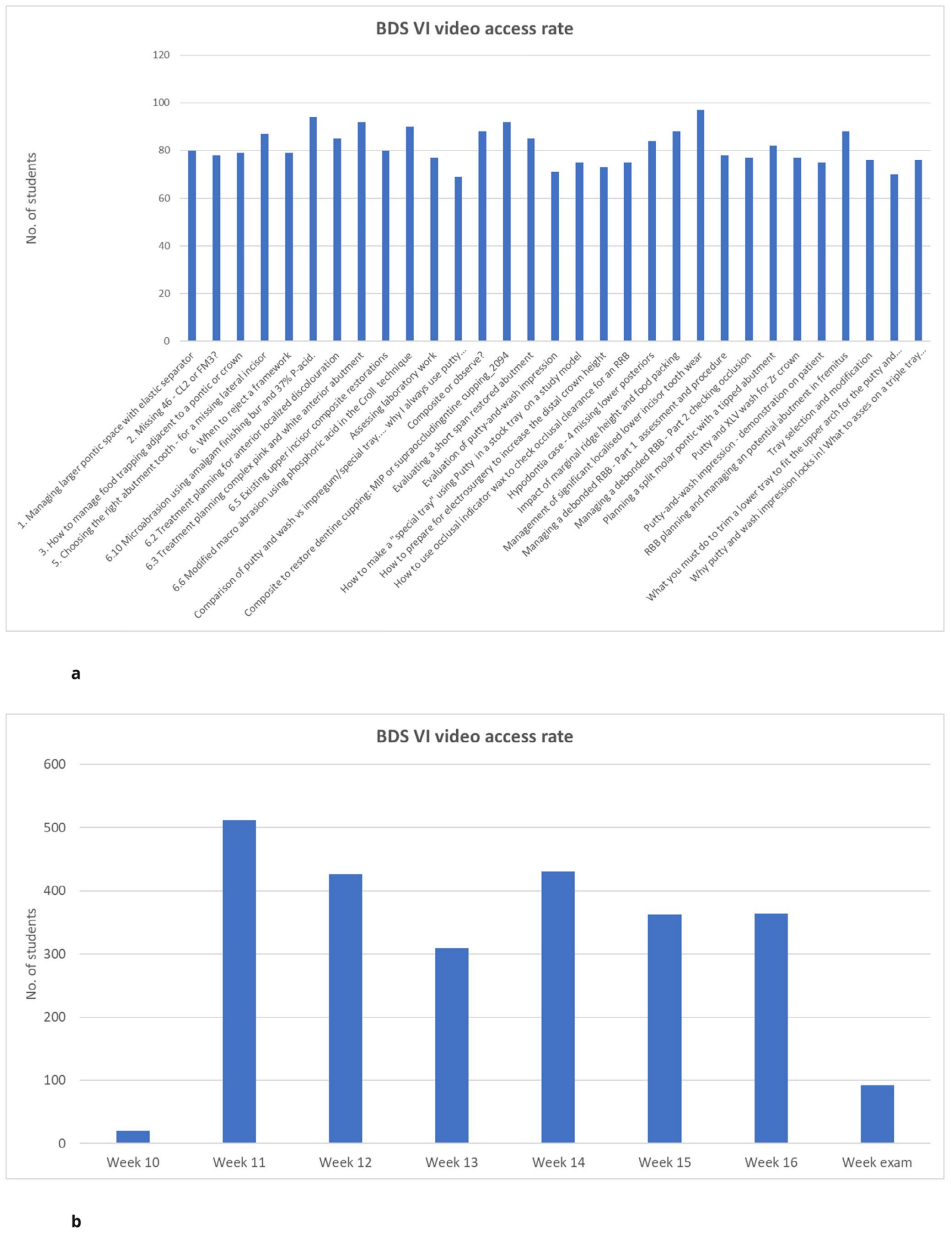
Access rate to the videos listed for the exam by the BDS VI students. (a) Number of viewings by video. (b) Number of viewings according to the week.

According to data on online video content consumption, all the videos were viewed by students (Figure 3a). However, there was a noticeable peak in views during the week that the exam scope and video list were announced, with over 500 views recorded. Views remained steady at 300–450/week until the week of the exam (Figure 3b).

#### 3.2.4 Generalisable

The students found this method of assessment to be useful and generalisable to other fields of study. They reported that using videos for learning could be valuable in different disciplines like ‘oral and maxillofacial surgery because we don't have much exposure. We haven't tried this surgery …’ and in ‘cariology specialty … orthodontics as well, … I'm thinking endodontics might be suitable as well’ (Student 5). ‘Almost every actually’ (Student 1).

Students reported a positive experience in comparison to other assessments. They described being happy about the scope of the exam notice, felt less stressed and were motivated to learn more ‘I really enjoyed this. This style of exam actually. It's very different from the ones that we used to have. Like, we were never really given like videos to really watch and to apply in our learning’ (Student 9).

### 3.3 Video as a Learning Medium

#### 3.3.1 Enjoyable

Students reported that watching videos was an enjoyable mode of learning in comparison to ‘just looking at the words’ (Student 5), ‘it's quite boring to just look at the fixed partial denture manual’ (Student 12). They described it as ‘kind of a comfort that we have something to watch’ (Student 5). Students also shared that having access to videos made them ‘more motivated to review our knowledge on all these topics’ (Student 12).

#### 3.3.2 Affordance

The video affords learning experiences that are not possible with textbooks or lecture notes and so students can visualise in detail how they can apply the knowledge in the videos to their own patients.

You can actually see how it's done rather than just read about it and, you know, imagine it in your head (Student 2).

## 4 Discussion

The aim of our study was to determine students' perception of an assessment that uses a novel approach for the evaluation of knowledge and cognitive skills in a summative assessment. It used an innovative type of clinical video that captures clinical learning experiences of other students to facilitate procedural understanding, diagnosis, problem‐solving and clinical decision‐making. The list of video learning resources was released to students to drive the students' learning to the online video resources to engage them with the content and allow an assessment directed at higher‐order thinking skills.

The online video resources facilitated flexible learning with regard to time, preferred environment and learning modalities. Students adopted various learning strategies such as watching the videos in groups or reviewing them individually and making notes during or after watching the videos. In addition the online video resources allowed students to take screenshots of key moments in the video as visual notes and pause and review the video at their own pace. Such strategies have been previously observed to improve engagement among students [11]. The conceptual theory underpinning this is the learner control principle which has been suggested to afford an active and constructive processing of information as well as enhancing motivation to learn. When students have control over the content, they can shape their own learning experience and actively build their knowledge from the materials, rather than being passive recipients of pre‐structured information. This approach is supported as a way to promote more effective learning [15, 16]. By taking an active role in directing their learning, students are more likely to be engaged and motivated and are better equipped to adapt to their individual preferences and needs.

Videos also afford the potential in shaping the focus of learning depending on their content and context. In a group of midwifery students, case‐based learning utilising clinical contextual video engaged students to pay more attention to the psychosocial aspect of the case compared to biomedical aspects whereas the students who only experienced a paper version of the case focused more on the biomedical than psychosocial aspects [17]. This demonstrates that video can offer different cognitive engagement compared to traditional paper‐based learning and allows opportunities for different thought processes and potential outcomes.

Dentistry is a highly stressful course for student learning, with assessments being perceived as particularly stressful events. Stress can negatively impact student performance, with stressed students reporting lower exam results than non‐stressed students [18]. One of the benefits of the current summative assessment was the fact that there was a well‐defined scope of learning which was communicated to students in advance. This information of what is required helped students to not only prepare more effectively for the exam but also reduce their stress levels. Reducing the stress associated with exams could offer significant benefits for students. By attenuating stress levels, students are more likely to perform better, maintain their well‐being and achieve success in their academic pursuits [18, 19].

However, the prescribed scope of learning may mean that students may neglect other parts of the course learning outcomes. Therefore, a balance must be found between the scope of knowledge assessed and limiting adverse examination stress, this may be achieved by expanding this type of assessment to different disciplines and increasing the frequency of assessment by using an assessment for learning approach to reduce stress and broaden the scope of knowledge assessed. Strategies such as expanding the use of formative assessments and providing briefings on exam formats have been suggested in the literature as effective means to reduce stress and enhance learning outcomes [20].

Given the scope of the clinical subject matter, students reported being stimulated to further learning in the relevant content areas. In particular, the vicarious learning dialogue videos demonstrated authentic teaching and learning moments in the context of clinical problems based on peer experiences. These chairside discussions with a clinical teacher covered a broad range of topics where students reported learning new skills and knowledge. Therefore, this vicarious experience allows students to gain insights from peers as well as broadening their clinical experience to new or diverse cases they may not have been exposed to during their clinical training. Such exposure to new learning content helps students develop a deeper understanding of the subject matter and allows them to apply their newly acquired knowledge as well as problem‐solving and clinical decision‐making skills in future contexts [10, 13].

Observing other students' clinical experiences also can facilitate students learning new content and skills as well as revising old ones. Observing peers in videos has been shown to be effective in other disciplines. In physiotherapy, students watched videos of peers taking a practical examination which consists of mock clinical consultations with two clinical cases. This helped peers to improve content understanding and reduce stress as in the current study and was reported to be effective and enjoyable for their learning [21].

This study emphasises the way that assessment drives learning as students reported watching all the prescribed video resources. This is supported by a similar study that observed a high consumption rate of vicarious learning dialogue videos prior to clinical competence assessment [12].

Students found this type of exam question to be fair, enjoyable and clinically relevant to their learning and application. This assessment provided students with an opportunity to show their clinical knowledge in a somewhat similar format to an OSCE, in that it was directed at higher‐order thinking in relation to clinical skills. However, this is still a written exam with a focus on higher‐order thinking skills and may offer a possible efficient alternative to OSCEs when evaluating higher‐order thinking skills. However, that does not allow evaluation of the whole clinical performance or interaction with the student.

While the study provides valuable insights into cognitive skills assessment, there are some limitations. For instance, the self‐selection process used in the interview may influence the findings; however, a data saturation process was followed. Alternatively, a survey could be undertaken. Also, this study focused on a single subject area within a larger exam paper, consisting of eight different sections. Two written papers were used, with each section addressing a different clinical subject. To address these limitations, future studies could employ a survey to broaden feedback from students or create a new type of exam paper based on such vicarious learning dialogue videos that may facilitate clinical decision‐making and problem‐solving. Despite these limitations, these findings can serve as a starting point for future research aimed at improving assessment methods and fostering enhanced cognitive skills such as clinical decision‐making and problem‐solving.

## 5 Conclusion

This new approach to assessment based on content and process was found to be beneficial for students' preparation for a summative assessment with regard to reduced student stress, facilitating a more focused learning approach and learning new content and higher‐order thinking skills. There was virtually complete engagement with the prescribed video content, as evidenced by the learning analytics and students reported enjoying this assessment method compared to traditional ones.

## Conflicts of Interest

The authors declare no conflicts of interest.

## Supporting information


**Appendix S1.** List of the 42 videos by 4 domains with time of each video.
**Appendix S2.** Criteria for reporting qualitative research (COREQ).

## Data Availability

The data that support the findings of this study are available on request from the corresponding author. The data are not publicly available due to privacy or ethical restrictions.
