# Supplementary material for: Impact of Clinical Video Scenarios Used for a Summative Exam to Facilitate Learning
Source: Eur J Dent Educ. 2024 Dec 2;29(1):175–85. doi: 10.1111/eje.13050 (PMC11745209; doi:10.1111/eje.13050)
Supplement: Supplementary file 1 — Appendix S1. List of the 42 videos by 4 domains with time of each video. Appendix S2. Criteria for reporting qualitative research (COREQ). [file EJE-29-175-s001.docx]

**Appendix S1** : List of the 42 videos by 4 domains with time of each video.

| 1. Resin-Bond bridges |  |
| --- | --- |
| 1.1 Treatment Planning & Design Problems |  |
| Managing larger pontic space with elastic separator | 06:33 |
| Missing 46 – CL2 or FM3? | 03:08 |
| How to manage food trapping adjacent to a pontic or crown | 03:53 |
| Choosing the right abutment tooth - for a missing lateral incisor | 05:47 |
| When to reject a framework | 03:13 |
| Treatment planning RBB framework with a large buccal composite | 05:26 |
| Dogmas or poor pontic design! Posterior CL2 RBB | 05:08 |
| Split molar pontic design | 04:58 |
| Four lower arch CL2 RBBs and porcelain laminate veneers to reduce space | 10:06 |
| No preparation RBB | 03:52 |
| Simple but. a little complex – 41 extracted | 03:31 |
| 1.2 Assessing laboratory Work |  |
| Assessing laboratory work | 03:46 |
| Impact of marginal ridge height and food packing | 03:30 |
| Evaluating a short span restored abutment | 02:50 |
| 1.3 Clinical assessment |  |
| How to use occlusal indicator wax to check occlusal clearance for an RBB | 02:31 |
| How to prepare for electrosurgery to increase the distal crown height | 05:32 |
| RBB planning and managing an potential abutment in fremitus | 07:13 |
| 1.4 Managing Deboned RBBS |  |
| Managing a debonded RBB – Part 1 assessment and procedure | 04:25 |
| Managing a debonded RBB – Part 2 checking occlusion | 01:30 |
| Management of de bonded RBB retainer posterior localized tooth wear, 20-year-old FM - RBB | 06:02 |
| 1.5 Case Discussion All Zirconia RBBs |  |
| Hypodontia case – 4 missing lower posteriors | 10:45 |
| Planning a split molar pontic with a tipped abutment | 03:43 |
| 2. Crowns and fixed partial dentures |  |
| 2.1 Impression |  |
| Tray selection and modification | 01:02 |
| Putty and wash impression – demonstration on patient | 04:39 |
| Evaluation of putty and wash impression | 02:07 |
| How to make a “special tray” using Putty in a stock tray in a study model | 04:27 |
| Why putty and wash impression locks in! what to assess on a triple tray impression | 05:11 |
| What you must do to trim a lower tray to fit the upper arch for the putty and wash technique | 03:23 |
| Comparison of putty and wash vs impregum/special tray…. why I always use putty and wash | 04:04 |
| Putty and XLV wash for Zr crown | 04:38 |
| 3. Aesthetics |  |
| Median diastema correction – Diagnostic wax-up, planning, intraoral mock-up | 08:51 |
| Treatment planning for anterior localized discoloration | 05:41 |
| Treatment planning complex pink. and white anterior abutment | 10:29 |
| Existing upper incisor composite restorations | 02:10 |
| Modified macro abrasion using phosphoric acid in the Croll technique | 05:39 |
| Microabrasion of white and brown spots | 04:12 |
| Microabrasion using amalgam finishing bur and 37% P-acid | 04:14 |
| 4. tooth wear |  |
| 4.1 Supra occluding restorations/Dahl principle |  |
| Management of significant localized lower incisor tooth wear | 06:59 |
| Direct composite to restore 36,46 tooth surface loss | 07:56 |
| Management of de bonded RBB retainer posterior localized tooth wear, 20-year-old FM – RBB | 06:02 |
| 4.2 Conservative management of tooth wear |  |
| Composite to restore dentine cupping: MIP or supraoccludingntine cupping_2094 | 03:49 |
| Composite or observe? | 04:08 |

**Appendix S2:** Criteria for reporting qualitative research (COREQ)

| **Personal Characteristics** |  | Reported |
| --- | --- | --- |
| 1. Interviewer/facilitator | Which author/s conducted the interview or focus group? | ✔ |
| 2. Credentials | What were the researcher’s credentials? E.g. PhD, MD | ✔ |
| 3. Occupation | What was their occupation at the time of the study? | ✔ |
| 4. Gender | Was the researcher male or female? | ✔ |
| 5. Experience and training | What experience or training did the researcher have? | ✔ |
| **Relationship with participants** |  |  |
| 6. Relationship established | Was a relationship established prior to study commencement? | ✔ |
| 7. Participant knowledge of the interviewer | What did the participants know about the researcher? e.g. personal goals, reasons for doing the research | ✔ |
| 8. Interviewer characteristics | What characteristics were reported about the interviewer/facilitator? e.g. Bias, assumptions, reasons and interests in the research topic | ✔ |
| **Theoretical framework** |  |  |
| 9. Methodological orientation and Theory | What methodological orientation was stated to underpin the study? e.g. grounded theory, discourse analysis, ethnography, phenomenology, content analysis | ✔ |
| **Participant selection** |  |  |
| 10. Sampling | How were participants selected? e.g. purposive, convenience, consecutive, snowball | ✔ |
| 11. Method of approach | How were participants approached? e.g. face-to-face, telephone, mail, email | ✔ |
| 12. Sample size | How many participants were in the study? | ✔ |
| 13. Non-participation | How many people refused to participate or dropped out? Reasons? | ✔ |
| **Setting** |  |  |
| 14. Setting of data collection | Where was the data collected? e.g. home, clinic, workplace | ✔ |
| 15. Presence of non-participants | Was anyone else present besides the participants and researchers? | ✔ |
| 16. Description of sample | What are the important characteristics of sample? e.g. demographic data, date | ✔ |
| **Data collection** |  |  |
| 17. Interview guide | Were questions, prompts, guides provided by the authors? Was it pilot tested? | ✔ |
| 18. Repeat interviews | Were repeat interviews carried out? If yes, how many? | x |
| 19. Audio/visual recording | Did the research use audio or visual recording to collect the data? | ✔ |
| 20. Field notes | Were ﬁeld notes made during and/or after the interview or focus group? | x |
| 21. Duration | What was the duration of the interviews or focus group? | ✔ |
| 22. Data saturation | Was data saturation discussed? | ✔ |
| 23. Transcripts returned | Were transcripts returned to participants for comment and/or correction? | x |
| **Data analysis** |  |  |
| 24. Number of data coders | How many data coders coded the data? | ✔ |
| 25. Description of the coding tree | Did authors provide a description of the coding tree? | x |
| 26. Derivation of themes | Were themes identiﬁed in advance or **derived from the data?** | ✔ |
| 27. Software | What software, if applicable, was used to manage the data? | ✔ |
| 28. Participant checking | Did participants provide feedback on the ﬁndings? | x |
| **Reporting** |  |  |
| 29. Quotations presented | Were participant quotations presented to illustrate the themes / ﬁndings? Was each quotation identiﬁed? e.g. participant number | ✔ |
| 30. Data and ﬁndings consistent | Was there consistency between the data presented and the ﬁndings? | ✔ |
| 31. Clarity of major themes | Were major themes clearly presented in the ﬁndings? | ✔ |
| 32. Clarity of minor themes | Is there a description of diverse cases or discussion of minor themes? | ✔ |
